# The diastolic shock index works… but, what is it?

**DOI:** 10.1186/s13613-020-00720-5

**Published:** 2020-08-03

**Authors:** Rafael Dalmau

**Affiliations:** Department of Anesthesiology, Hospital Español de Rosario, Rosario, Argentina

Editor

The recent study by Ospina-Tascón et al. [1] presents a novel hemodynamic index—the “diastolic shock index” (DSI), defined as the ratio of heart rate (HR) and diastolic arterial pressure (DAP)—distinguishing itself from the “classical” and “modified” shock indices [2], which are based on the systolic and the mean arterial pressure, respectively.

On the basis that “DAP depends [directly] on vascular tone and [inversely] on the duration of the cardiac cycle [i.e., the reciprocal of HR]” [1], the authors targeted the DAP, with the following goals: to assess the state of the vascular tone; to predict the severity and clinical outcomes of patients undergoing septic and other vasodilatory shock states; and to guide early vasopressor therapy.

With this rationale in mind, there are three matters that, given the intuitive and empirical nature of this index, might have been overlooked in its formulation. The first two address the meaning of the ratio between HR and arterial pressure (AP), and the third one is exclusively devoted to the DSI.

The word “index” has more than one meaning, but, as a general rule, an index is supposed to relate quantities independent of each other, in order to obtain additional and different information from that given separately by the individual quantities 1. More precisely, mean arterial pressure − mean central venous pressure = CO × TPR.

In the case of the shock indices, the variables involved—HR and PA—are already interrelated by a preexisting function, so taking their ratio is redundant. With PA = CO × total peripheral resistance (TPR)^2^ and CO = HR × stroke volume (SV), we can rewrite PA = HR × SV × TPR; in other words, the ratio HR/PA is already implied in the functional relationship between them.

Secondly, there is the principle of dimensional consistency, by which two entities with different dimensions cannot represent the same thing. If the ratio of HR and AP is supposed to represent something else (here, certain “state” of the cardiovascular system in shock), the shock index should have dimensions (and the corresponding units) of that quantity.

If the DSI, in particular, is to represent the vascular tone [1], then the units of the quantity representing it should be of [Time^−1^] × [Pressure^−1^], which, evidently do not correspond to any mechanical property of the vascular wall related to its “tone” (whether it is wall tension, arterial resistance, or arterial compliance), which leads to the third and final point.

The direct link, or dependence, between DAP and arterial vasomotor tone is well known in the literature, and is the basis for the present DSI. However, there seems to be no apparent reason as to why the DAP should *preferentially* reflect the vascular tone, or more than the systolic pressure does. In other words, both the systolic *and* diastolic pressures are determined by vascular factors.

This matter is not miscellaneous, as with no basis for linking DAP specifically to arterial tone there would be no justification for distinguishing the DSI from the classical or the modified shock indices. A plausible answer to this question may be found in simple “Windkessel” theory 3:

$$P\left( t \right) = P\left( {t_{d} } \right)e^{{\frac{{ - \left( {t - t_{d} } \right)}}{{\left( {RC} \right)}}}},$$

where the exponential pressure decay from the start of diastole (*t*_*d*_) is governed by the time constant “*RC*” (i.e., arterial resistance times compliance) [3].

In this way, as the DAP is mainly determined by the diastolic time constant *RC*, one could interpret it as being representative of the arterial tone, while the dependence of systolic arterial pressure on the arterial *RC* is more directly confounded by other parameters—namely, heart function parameters—than the DAP is.

Finally, these observations are not intended to disregard the potential benefits of implementing the DSI as a statistical predictor of some event related to septic shock. Perhaps, its success is explained by the fact that the two quantities somehow interrelated, one linked to the heart and the other linked to the vasculature, are simultaneously analyzed reflecting the interaction between the two systems.

## Data Availability

Not applicable.

## Abbreviations

AP: Arterial pressure
C: Compliance
CO: Cardiac output
DAP: Diastolic arterial pressure
DSI: Diastolic shock index
*e*: Base of the exponential function
HR: Heart rate
*P*: Pressure
R: Resistance
TPR: Total peripheral resistance
*t*: Time
*t*_*d*_: Time at the start of diastole

## Footnotes

1 For instance, the “cardiac index” relates CO to body surface area, yielding a *new* quantity relating heart performance to the size of the individual, and which corresponding units are consistent with the new information which is not found in the definition of either of the two separate magnitudes. Likewise, if the ratio of HR/DAP is to represent the vascular tone, the units should be compatible.

2 More precisely, mean arterial pressure − mean central venous pressure = CO × TPR.

3 For simplicity, higher-order elements of the Windkessel model, as well as complex phenomena of arterial wave reflection, are neglected in the present consideration.
